# A Recursive Algorithm for Indoor Positioning Using Pulse-Echo Ultrasonic Signals

**DOI:** 10.3390/s20185042

**Published:** 2020-09-04

**Authors:** Salvatore A. Pullano, Maria Giovanna Bianco, Davide C. Critello, Michele Menniti, Antonio La Gatta, Antonino S. Fiorillo

**Affiliations:** 1 Department of Health Sciences, University “Magna Græcia” of Catanzaro, Viale Europa, 88100 Catanzaro, Italy; pullano@unicz.it (S.A.P.); mg.bianco@unicz.it (M.G.B.); critello@unicz.it (D.C.C.); menniti@unicz.it (M.M.); 2 LRO—London Research Organization, 207 Regent Street, London W1B3HH, UK

**Keywords:** ultrasonic transducers, time of flight estimation, pulse-echo technique, ferroelectric films, piezopolymer

## Abstract

Low frequency ultrasounds in air are widely used for real-time applications in short-range communication systems and environmental monitoring, in both structured and unstructured environments. One of the parameters widely evaluated in pulse-echo ultrasonic measurements is the time of flight (TOF), which can be evaluated with an increased accuracy and complexity by using different techniques. Hereafter, a nonstandard cross-correlation method is investigated for TOF estimations. The procedure, based on the use of template signals, was implemented to improve the accuracy of recursive TOF evaluations. Tests have been carried out through a couple of 60 kHz custom-designed polyvinylidene fluoride (PVDF) hemicylindrical ultrasonic transducers. The experimental results were then compared with the standard threshold and cross-correlation techniques for method validation and characterization. An average improvement of 30% and 19%, in terms of standard error (SE), was observed. Moreover, the experimental results evidenced an enhancement in repeatability of about 10% in the use of a recursive positioning system.

## 1. Introduction

Over the years, ultrasonic technology has been applied in variegated fields ranging from underwater acoustics [1,2], medical imaging [3] and biomedical devices [4,5]. Apart from the above, indoor localization systems have reached a widespread consensus as they are inexpensive, space-saving and less prone to interference due to environmental light or heat sources [6,7,8,9]. In-air ultrasounds were amply investigated to retrieve information about unstructured environments in 3D tracking and motion detection [6,7,10,11,12,13,14,15]. Although most technologies (infrared radiation, radio frequency, artificial vision) are currently developed and commercialized, systems based on ultrasounds can be realized with simple hardware [10,16], combining multiple coplanar transmitters [17] or in association with multiple receivers [18], easily achieving a sub-mm resolution. 

However, the performances of the 3D ultrasonic positioning system can be significantly improved by working on hybrid technologies or a novel algorithm [12,19].

Conversely, in-air ultrasounds, which usually range from 30 to 120 kHz, are poorly suitable in the case of long distances and for the most sophisticated fine-grained local positioning systems (LPSs), because of the signal wavelength, and the wide lobe of irradiation of the available transducers [14,20]. Recent literature reports different attempts to overcome the limitation of commercially available transducers in terms of bandwidth, sensitivity and directivity, by introducing novel geometries and by the optimization of acoustic wave propagation [7,21,22,23,24,25,26]. LPSs are usually realized with a combination of multiple transmitters/receivers, properly positioned around the target area. The emitted signal and the received echo provide different basic information, such as the receiving object distance, through a time of flight (TOF) estimation or other information about target characteristics, as in the case of bio-inspired echolocation systems [27,28]. The simplest and most common way to detect TOF echo signals is the threshold method, in which the detection occurs when a signal crosses a predetermined threshold [29,30,31]. It is generally characterized by a lower accuracy introduced by the sampling frequency, low signal-to-noise ratio (SNR), and difficulty in setting an optimized threshold. The introduced delay is generally nonconstant, resulting in a variable offset error. Another widely used technique involves the cross-correlation function to estimate the TOF of pulse-echo signals by varying the time observation point [31,32,33]. The latter is also exploited in natural bio-sonar, in a neural approach for calculating the temporal correlation between pulse and echo [29,34,35]. Other approaches exploit artificial intelligence techniques as a probabilistic algorithm, artificial neural networks, k-nearest neighbor or support vector machine to evaluate the position of an object and improve automatized learning [36,37].

Although for most applications they provide a sufficient level of accuracy, they are inherently sensitive to SNRs, distortion and other factors such as fluctuations of sound velocity and the proximity of other objects. In this paper, a modified cross-correlation technique, based on pulse-echo analysis, is investigated for a recursive TOF evaluation. The transmitted pulse and the echo are generated by curved polyvinylidene fluoride (PVDF) transducers previously investigated for robotic applications, characterized by a low quality factor and high coupling in air [7,28]. The technique is based on a recursive cross-correlation analysis and the use of a template signal as a reference. The TOF is evaluated with respect to a calibrated echo signal, resulting in an improved accuracy and repeatability during continuous target monitoring. The proposed approach is directed to the development of a new algorithm which, together with the advancements in sensors technologies, can provide improvements in real-time driver monitoring and behavior, especially if integrated with complementary technologies (e.g., alcohol monitoring, fatigue recognition systems).

## 2. Materials and Methods

### 2.1. Ultrasound Sensors

The application of ultrasonic sensors in determining the x, y, z coordinates of an object in a working space (e.g., cockpit, robot space) can be used complementarily with optical systems or alone as a valid alternative to optical methods with a reduced sensitivity to noise, dust, lighting conditions, etc. [38]. In SONAR (Sound NAvigation and Ranging) systems, the resolution can be correlated with the spectral content of the received signals. The radial resolution in a sonar system is a function of the bandwidth, whereas the azimuth resolution is a function of the system opening [39,40]. In air, the time of flight of ultrasonic waves at different frequencies can be considered almost the same; thus, the resolution is limited by the data acquisition and processing. The propagation medium introduces an attenuation which depends on different factors like beam dispersion, hysteresis, friction losses and the viscosity of the medium. Moreover, attenuation increases with frequency, which can alter the reflected wave [41]. External noises, such as turbulence, vibrations and the noise due to the electronics used, also affect the received echo travelling in a medium. By only taking into account the air viscosity, the enlargement of the acoustic beam mainly depends on the displacement with respect to the source and the attenuation of the medium according to the Lambert–Beer law.

**Figure 1:**
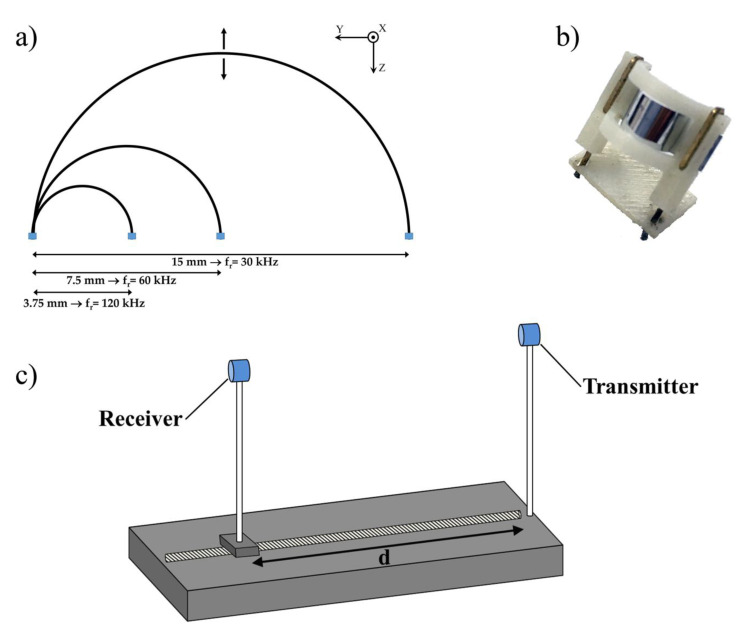
Schematic of hemicylindrical geometries resonating at 30, 60 and 120 kHz, respectively (**a**). A 60 kHz fabricated transducer (**b**) and Scheme of the experimental setup for the time of flight (TOF) evaluation between transmitter and receiver (**c**).

Obviously, depending on the specific application and frequency of the system, it is always desirable to improve the resolution, in order to reduce ambiguity during target positioning and tracking. Bimodal transducers can result in a worse performance in terms of the SNR at the input of the receiver. In some cases, multiple unimodal transducers are thus preferred, in order to achieve an electrical and mechanical decoupling. Previously developed ultrasonic transducers, based on the ferroelectric properties of PVDF, were investigated for robotic applications. The hemicylindric geometry has been theoretically and experimentally investigated in the range between 30 and 120 kHz [3,42]. The transducer was made with a strip of PVDF with a thickness of 28 μm, a width of 5 mm and a length which depended on the specific resonance frequency (*f_r_*). The strip was metallized on both faces, with about 200 nm of aluminum and clamped on the short side in order to achieve a hemicylindrical geometry. The operating principle was based on the conversion of longitudinal motion into radial vibrations due to the clamped extremities (caused by the alternating voltage applied between the electrodes) allowing the generation of radial acoustic waves in the anterior (concave) and posterior (convex) sides [7,14]. The resonance frequency was inversely proportional to the bending radius and, therefore, could be easily manipulated by varying the curvature. Due to the very low-quality factor of the transducer (Q about 12), the signal is characterized by a broad spectrum. Deviation of the resonance frequency (~5%) can be observed with respect to the theoretical value due to assembly defects (not perfectly hemicylindrical, nonparallel electrode shapes), as well as parasitic resistances created during the realization of the external electrodes (e.g., silver paste, pressure contacts). Figure 1a reports the effective dimensions of few representative sensors and the related diameters, while Figure 1b shows the supporting structure used to maintain the geometry, the curved PVDF film, and the external contact.

The experimental set-up was composed by two unimodal 60 kHz PVDF transducers, one transmitter and one receiver, facing each other at a variable distance *d* (Figure 1c). The transmitter was characterized by a sound pressure level (SPL) of 105 dB, considering a reference pressure of 20 µPa (0 dB) at 0.3 m. The receiver, instead, had a sensitivity of −80 dB, considering a reference sensitivity of 10 V/Pa (0 dB). Both unimodal transducers had a bandwidth of 5 kHz [25,43,44]. The acoustic beam was generated by driving the PVDF transmitter with a pulse of 10 sinusoidal cycles at 60 kHz, with a peak-to-peak voltage of 2V (Tektronix AFG3102), amplified by 36.5 dB through a power amplifier stage. The echo conditioning circuit was composed by a low noise amplifier, a band pass filter and a further amplifier stage. The PVDF receiver was shunted by a couple of diodes with the purpose of protecting the low-noise amplification stage from excessive amplitude voltage signals that the transmitting stage or other noise sources could capacitively induce. The ultrasonic beam was characterized according to the Institute of Electrical and Electronics Engineers (IEEE) international standard by means of intensity parameters. The spatial peak-temporal peak intensity (I_sptp_), spatial peak time average (I_spta_) and spatial peak pulse average (I_sppa_) were determined over a plane 300 mm from the ultrasonic transmitter, using a wide-band system composed by a conditioning amplifier (Brüel and Kjaer NEXUS 2692-C) and a ¼” free-field microphone, 4 to 100 kHz, 200 V polarization (Brüel and Kjaer, Type 4939). The ultrasonic signal was detected by the PVDF receiver, conditioned, and the was voltage recorded by a digital oscilloscope (Tektronix DPO 3054) [45]. 

### 2.2. Monitoring Routine

**Figure 2:**
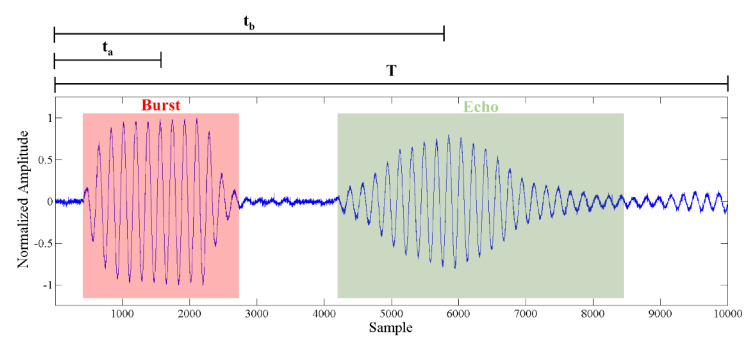
Pulse-echo signal (60 kHz) transmitted and received through a couple of hemicylindrical polyvinylidene fluoride (PVDF) transducers.

The transmitter was driven with a sinusoidal burst with a frequency *f_r_*, allowing the generation of an acoustic signal, which was propagated toward the target (receiver), then transduced and conditioned, obtaining a voltage profile as shown in Figure 2. The cross-correlation gives a measure of waveform similarities while shifting one of them onto the other. Since the cross-correlation of white noise approaches to zero, the cross-correlation was inherently characterized by noise reduction. Moreover, in order to reduce the frequency and phase errors, the signal envelope was obtained before starting the signal processing [19]. Given two digital sequences y_P_(kT^S^) and y_E_(kT_S_) of the pulse and echo signals, respectively, where T_S_ is the sampling time, the cross-correlation is given by:

(1) $$X_{C}=\sum_{-\infty }^{+\infty }y_{P}\left(kT_{S}\right)\cdot y_{E}\left(kT_{S}+nT_{S}\right)$$

The estimation of the time delay between the two sequences was evaluated trough the maximum of Xc. Let us now consider the signal as shown in Figure 2, used to represent the transmitted pulse (red shaded area) and the received echo (green shaded area). In the time domain the differences between the maximum of the echo signal (t_b_) and the related pulse transmission time (t_a_) represents the time elapsed between ultrasonic source transmission and echo reception. 

**Figure 3:**
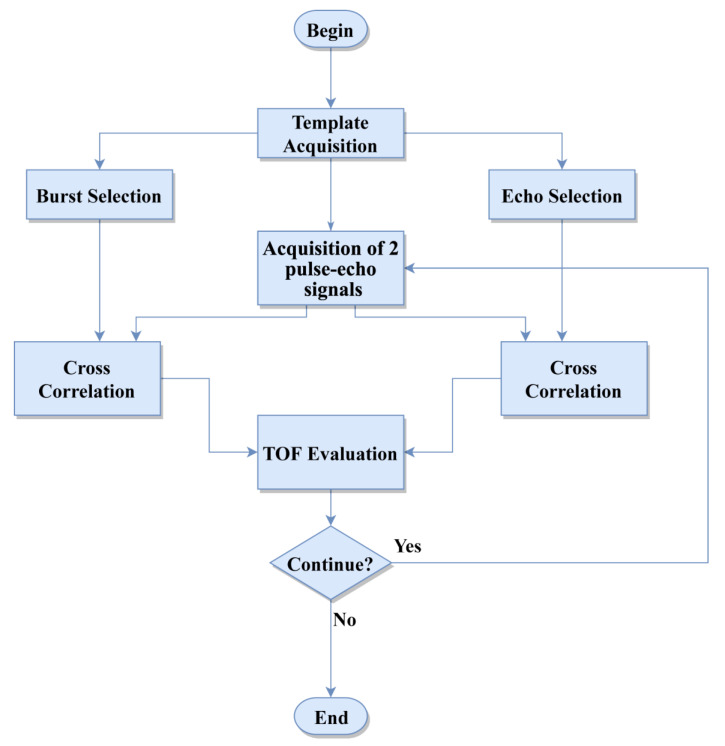
Transmission and reception model of the setup.

The distance was then computed by taking into account the sound velocity in air (*d = TOF·v*). Even though variable (influence of temperature, humidity, etc.), the sound velocity in air can be modeled with good approximation by *v = 20.555·√T,* where *T* is the temperature in Kelvin, to take into account the environmental conditions [46,47]. Since the time reference is used in signal acquisition, an accurate pulse-echo acquisition is necessary. Synchronization can be inherently affected by frequency errors (i.e., nonconstant errors) and in case of multiple reference signals these errors can affect each other. A time shift can be observed also in the case of a single reference signal used to synchronize transmission and reception. These synchronization errors are due to different factors, such as local temperature random errors. This means that the TOF is affected by smaller variations happening continually (i.e., time shift of the pulse and echo maximum t_a_ and t_b_). As shown in the flowchart (Figure 3), the processing technique starts with the acquisition of a pulse-echo signal at a given distance, named template signal, then the following steps were carried out: (i) selection of the pulse component *s_a_(t)*) and echo component (*s_b_(t)*, (ii) cross-correlation between two subsequent acquired signals and the pulse-echo, respectively, (iii) TOF evaluation and return to the acquisition of a new set of signals. 

**Figure 4:**
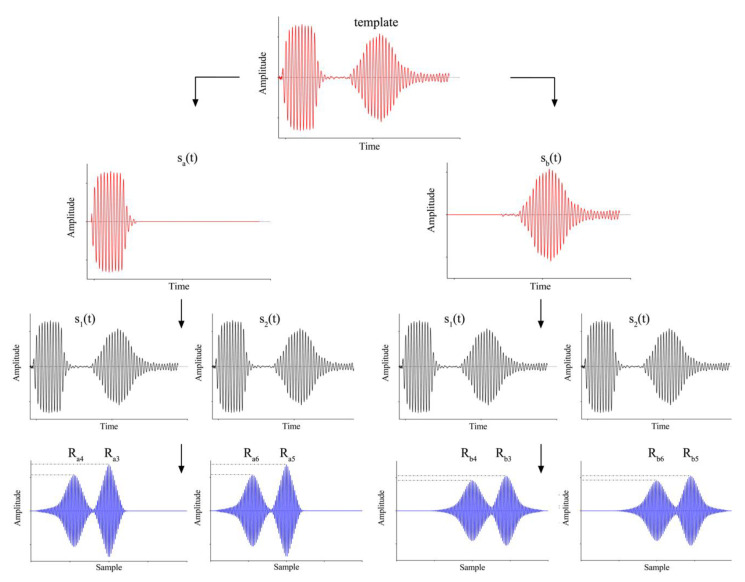
Ultrasound signal processing steps for TOF estimation through a modified cross-correlation-based technique: in red is reported the template signal, properly filtered in transmitter (*s_a_(t)*) and receiver (*s_b_(t)*); subsequently, two further acquired signals (*s_1_(t)* and *s_2_(t)* (in black)), were opportunely cross-correlated with the two templates, obtaining 4 cross-correlations (in blue); local cross-correlation maxima, related to the corresponding time shift for homologous (pulse–pulse) and nonhomologous (pulse-echo) signals, were obtained as reported in Table 1.

The use of a template signal allows for the referencing of all the cross-correlations to the same signal, which is expected to affect the accuracy of the TOF evaluation, especially on multiple cyclic transmissions/receptions. Moreover, in the proposed implementation, no envelope extraction was investigated. Considering two acquired pulse-echo signals, *s_1_(t)* and *s_2_(t),* shifted with respect to the template, similarly to what was done for the template signal (Figure 2), t_c_, t_d_, t_c2_, and t_d2_ indicate the referenced time at pulse, and the maximum echo time of *s_1_(t)* and *s_2_(t)*, respectively. The proposed TOF estimation through the modified cross-correlation technique according to the procedure previously described can be seen in Figure 4. 

**Figure 5:**
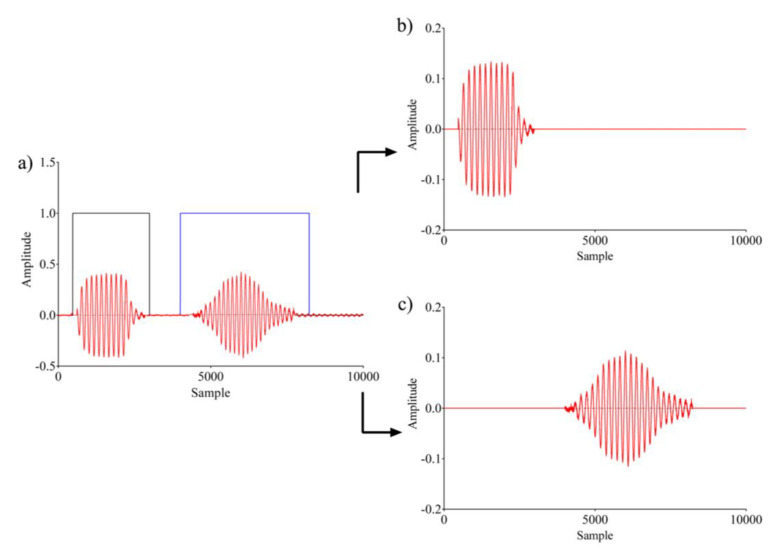
Representative signal windowing for maximum evaluation.

The cross-correlation between *s_a_(t)* and the template is in general characterized by two local maxima, the first, R_a1_, related to the maximum overlap between homologous (pulse–pulse) signals, while the second, R_a2_, related to the maximum overlap between nonhomologous (pulse-echo) signals (not shown in Figure 4). Similarly, the cross-correlation between s*_b_(t)* and the template evidenced other two local maxima, R_b1_ (pulse–pulse) and R_b2_ (pulse-echo). The same steps have been performed between the two template signals and *s_1_(t)*, *s_2_(t).* According to the proposed technique, 4 cross-correlations were evaluated providing multiple maxima, each one related to a specific time shift. Moreover, two more maxima were related to the cross-correlation of the template signal with *s_a_(t)* and *s_b_(t)*, which provides the calibrated initial position. A maxima evaluation of the pulse-echo and cross-correlation signals involves the selection of an appropriate Dirichlet window, with a time length *L*. The start and end of the window involves, firstly, the signal being rectified, binned (2 samples) and then set to a threshold (average value of the processed signal) with a window length overestimation of 10% (Figure 5). As each cross-correlation sample correlated with a specific time shift, the combination of information carried out by multiple cross-correlations can be used to retrieve the TOF related to the signals *s_1_(t)* and *s_2_(t)*. 

In Table 1, the local cross-correlation maximum is related to the specific time shift on which the proposed implementation is based. Therefore, the TOF evaluation is not affected by the choice of pulse–pulse or pulse-echo local maximum and, considering that the reference signal is the same, these times are expected to be more accurate than the times of flight evaluated through threshold and standard cross-correlation methods.

In this way, the distance between the transmitter and receiver can be evaluated by observing TOF increments with respect to the template signal (placed at a calibrated distance, related to TOF_0_). As we can verify, TOF_1_ and TOF_2_ can be alternatively obtained by analyzing the homologous or nonhomologous components of the cross-correlation. The reliability of the three methods were compared by the standard error SE = √(σ^2^/n), where σ^2^ is the sample variance and n is the sample size. Since a recursive evaluation is often required in positioning systems, investigations were performed by moving the receiver back and forth.

### 2.3. Experimental Validation

A set-up was fabricated in order to investigate the performance comparison between the threshold, standard and modified cross-correlation technique (Figure 1c). The system includes a threaded rod (M10 with a pitch of 1.5 mm), which is rotated by a 4-phase unipolar stepper motor (RS Components, Corby, UK) with a 7.5° step angle, 0.24 Nm holding torque and a positioning accuracy of 5%. The stepper motor has been driven by using a national instrument DAQ6015 board. A hemicylindrical ultrasonic transmitter was fixed solidly to the threaded rod, while the receiver had been placed at a reference position. 

**Figure 6:**
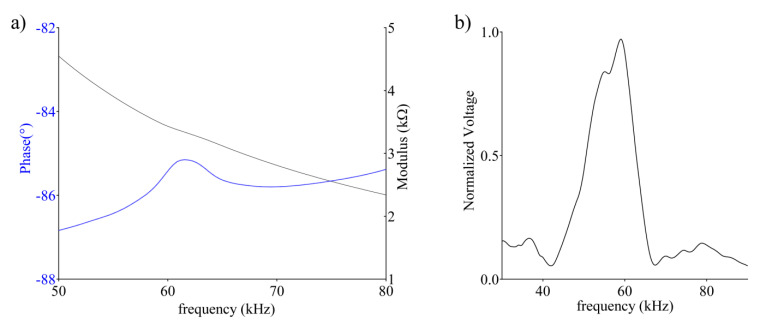
Impedance analysis (**a**) and frequency response (**b**) of a 60 kHz hemicylindrical PVDF transducer.

On the base of the number of steps and therefore the angular variation of the bar, the linear movement could be traced, apart from the errors due to the motor positioning and mechanical tolerances on the bar, which are assumed constant during the experimental evaluation. Considering the step angle and the pitch, the minimum longitudinal distance was evaluated by d*_L_*= *(p·ϕ)/360* (i.e., 0.03 mm). The supports, instead, gave the right height and the right alignment to the two sensors, so that the obstacles in the immediate vicinity did not create multiple reflections and, therefore, an echo signal with the presence of unwanted components. The impedance analysis and frequency response of the PVDF transducer evidenced the characteristic electric resonance feature (Figure 6a) and the bandwidth (Figure 6b) of the hemicylindrical sensor [48,49,50].

Starting from a predefined transmitter/receiver distance (set to 0.3 m), the stepper motor was driven to obtain a variable number of the turns from 1 (d*_L_* = 1.25 mm corresponding to 48 motor steps) up to 5 (d*_L_* = 6.25 mm corresponding to 240 motor steps) and the distance was maintained within 0.6 m. For each position, the signal acquisition was repeated four times for the statistical analysis. The effect of the pulse length was also investigated by changing the number of cycle *N* from 5 up to 15, corresponding to a pulse time duration of 83.3, 106.6 and 249.9 µs. The relationship between the actual distance and the relationship evaluated by the threshold, standard and modified cross-correlations were then compared. 

## 3. Results

Three excitation pulse signals were used to drive the PVDF transmitter. The stability of the excitation source was of ±1 ppm ±1 μHz, 0 to 50 °C, with expected amplitude variations < 10 mV. Therefore, the pulse was stable and controllable enough to be used as a reference signal for the cross-correlation method. The SNR was evaluated to be > 30 dB during all the acquisitions. As depicted in Figure 1c, the analyzed case is that of a transmitter facing a receiver with a separation distance controlled by the stepper motor. 

**Figure 7:**
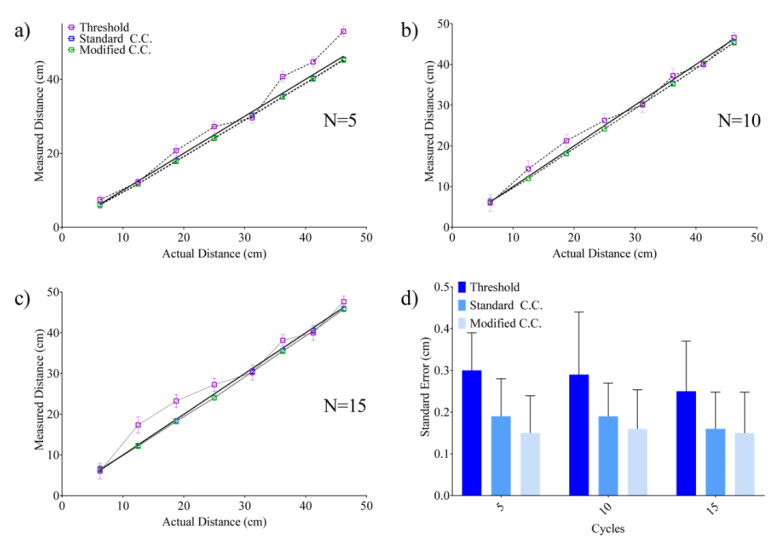
Comparison of distance evaluation used for a pulse length of (**a**) 5 sinusoidal cycles, (**b**) 10 sinusoidal cycles and (**c**) 15 sinusoidal cycles at 60 kHz. (**d**) Standard error in the distance evaluation.

The stepper motor was controlled by changing the turns and the TOF was subsequently evaluated with each method. Subsequently, the distance is computed taking into account the sound velocity in air by compensating the temperature fluctuation through a sensor, resulting in an uncertainty on the sound velocity of less than 0.05 m/s [46]. Figure 7a–c shows the comparison among threshold, standard and modified cross-correlation in the evaluation of TOF using a variable pulse length as previously reported, respectively. As expected, the standard and modified cross-correlation techniques performed better in terms of standard error (SE) and linearity with respect to the threshold technique (Figure 7d). 

**Figure 8:**
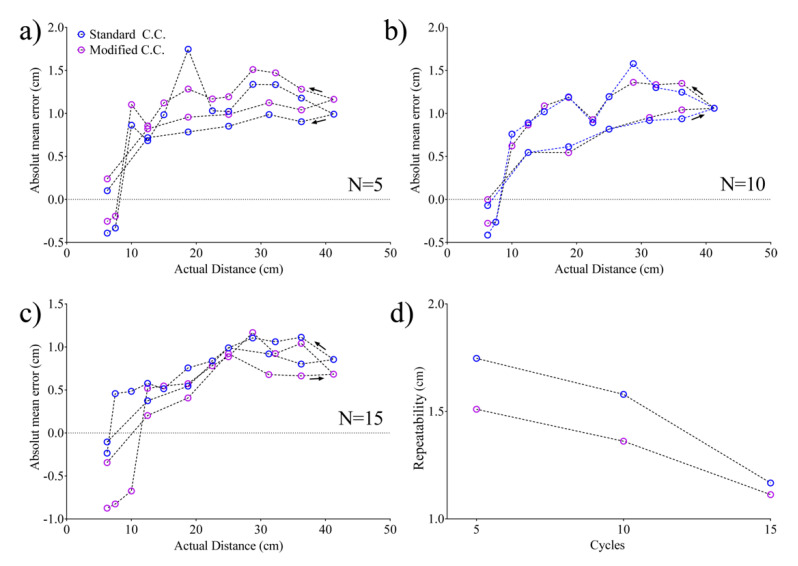
Repeatability evaluation through subsequent cycles of the TOF evaluation at increased/decreased distances using for a pulse length of (**a**) 5 sinusoidal cycles, (**b**) 10 sinusoidal cycles and (**c**) 15 sinusoidal cycles at 60 kHz. (**d**) Maximum repeatability error vs. pulse length.

The absolute mean errors reported in Figure 8a–c are representative of a target moving in a range of 40 cm, while the standard and modified cross-correlation techniques were used by varying the number of cycles *N*. Figure 8d reports the maximum error observed in the previously reported cases. In all cases, the results evidenced a nonlinear behavior, which however can be reduced by increasing the number of cycles (Figure 7d).

We additionally evaluated the computational time of both the standard and modified cross-correlations. In light of the results, the modified algorithm requires 70% of an additional computational load in the estimation of the TOF, which can be acceptable in most low frequency positioning systems.

## 4. Discussion

Based on the proposed technique, a template signal was evaluated as a reference signal for all the TOF evaluations in order to reduce errors due to synchronization that can be inherently affected by the range. The overall model is suitable in positioning systems working in a confined unstructured environment in which the distance between the transmitter and the target can be evaluated by observing TOF increments with respect to the calibrated position. In Figure 7d, it is clearly shown that a standard and modified cross-correlation exhibits a better performance than the threshold method. When increasing the number of cycles, no differences were highlighted between cross-correlation techniques, while the threshold method evidenced a deteriorated performance. Moreover, remarkable improvements with respect to the threshold technique are clearly observed with a reduction in SE in the order of 45%. Further improvements were also observed with respect to conventional cross-correlations which has been estimated in the order of 20%. This is mainly due to the use of a calibrated reference signal, which reduces the smaller variations that happen continually (i.e., time shift of the pulse and echo maximum). As previously highlighted, it is evident that this improvement is counterbalanced by a higher computational load. Moreover, no significant differences were observed by changing the pulse length in the range from 83.3 up to 249.9 µs, evidencing that it is possible to choose the pulse length in accordance with the requirements of the application without affecting the performances. Interestingly, the experimental results evidenced an enhancement in repeatability of about 10% by continuously changing the distance of the target back and forth, which means that it is possible to compensate for hysteresis-like behavior in the use of a recursive positioning system. Although the computational cost of the algorithm is higher than that of the compared techniques, it still guarantees the possibility of obtaining data in real-time for the specific application. In fact, an algorithm has been conceived for the monitoring and tracking of the driver, where a more accurate knowledge of driver dynamics can be used complementarily with other systems, providing shared information (e.g., the calibration of alcohol monitoring systems). The use of a single template signal for all the TOF evaluations can be advantageously applied in positioning systems based on multiple transmission/reception points, to reduce the time shift introduced by multiple reference signals. Moreover, the implementation of the combination of multiple data retrieved from a standard cross-correlation can reduce the time shift that can also be observed in the case of a single reference signal.

## 5. Conclusions

A study on a modified algorithm based on the cross-correlation technique for the evaluation of time of flight specifically designed for a recursive data evaluation was investigated. This proposed algorithm was implemented in MATLAB and a comparison with threshold and standard cross-correlation techniques was presented. The conventional resolution in SONAR is limited by the wavelength and, subsequently, different signal processing techniques, such as those based on cross-correlations. Of course, one of the ways to improve the overall performance of the system is to increase the ultrasound source frequency (i.e., a lower wavelength), and different SONAR systems were recently proposed in order to allow a frequency shift using wideband transducers. Obviously, ultrasonic attenuation in air dramatically increases as the frequency increases. The modified algorithm evidenced improvements with respect to both threshold and conventional cross-correlation techniques, with a reduction in the standard error of about 45% and 20%, respectively. On the other hand, an increase of 70% of computational load has been estimated in the evaluation of TOF. Nonintrusive on-board driver positioning can benefit the recursive nature of the algorithm and the electronic sensors investigated. 

## Figures and Tables

**Table 1:** Local cross-correlation maximum related to the corresponding time shift for homologous (pulse–pulse) and nonhomologous (pulse-echo) signals.

	Time	Pulse–Pulse Maxima	Pulse-Echo Maxima
R_a1_	T	R_a1_ − R_b2 =_ TOF_0_ R_a3_ − R_b4_ = TOF_1_ d_1_/v = (TOF_1_ − TOF_0_) R_a5_ − R_b6 =_ TOF_2_ d_2_/v = TOF_2_ − TOF_1_	R_b1_ − R_a2_ = TOF_0_+k k = t_b_ − t_a_ R_b3_ − R_a4_ = TOF_1_+k d_1_/v = (TOF_1_ + k) − (TOF_0_ + k) = TOF_1_ − TOF_0_ R_b5_ − R_a6_ = TOF_2_ + k d_2_ = (TOF_2_ + k) − (TOF_1_ + k) = TOF_2_ − TOF_1_
R_a2_	(T − t_d_) + t_a_		
R_b1_	(T − t_c_) + t_b_		
R_b2_	T − d		
R_a3_	T		
R_b3_	(T − t_c1_) + t_a_		
R_a4_	(T − t_d1_) + t_a_		
R_b4_	T − d_1_		
R_a5_	T		
R_b5_	(T − t_c2_) + t_a_		
R_a6_	(T − t_d2_) + t_a_		
R_b6_	T − d		

T = pulse-echo acquisition time; d = temporal distance between the reference signal and the shifted signal *s_1_(t)*; d_1_ = temporal distance between the reference signal and the shifted signal *s_2_(t)*; TOF= temporal distance between the shifted signals *s_1_(t)* and *s_2_(t);*
